# Factors associated with coverage of praziquantel for schistosomiasis control in the community-direct intervention (CDI) approach in Mali (West Africa)

**DOI:** 10.1186/2049-9957-2-11

**Published:** 2013-06-10

**Authors:** Abdoulaye Dabo, Boubacar Bary, Bourema Kouriba, Oumar Sankaré, Ogobara Doumbo

**Affiliations:** 1 Department of Epidemiology of Infectious Diseases, Faculty of Medicine, Pharmacy and Dentistry, UMI 3189, University of Sciences, Techniques and Technologies of Bamako, Box 1805, Bamako, Mali; 2 High Institute for Training and Applied Research, Box 475E, Bamako, Mali

**Keywords:** Schistosomiasis, Praziquantel, Community-directed intervention, Coverage rate, Diéma, Mali

## Abstract

**Background:**

Despite the progress made in the control of Neglected Tropical Diseases (NTD), schistosome and soil-transmitted helminth infections are far from being effectively managed in many parts of the world. Chemotherapy, the key element of all control strategies, is faced with some difficulties in terms of access to treatment. Our study aims to describe the factors involved in the success or failure of the community-directed intervention (CDI) approach through control programmes, which aims to achieve consistent high coverage at affordable and sustainable costs in endemic areas.

**Methods:**

The CDI approach was carried out from December 2007 to October 2008 in ten villages of the district of Diéma, Mali. At inclusion, each child part of the study’s sample was interviewed and submitted for a physical examination. The study focused on: data collection, treatment of the eligible population, evaluation of the treatment coverage, performance of community drug distributors (CDDs), and the involvement and perception of populations.

**Results:**

A total of 8,022 eligible people were studied with a mean coverage rate of 76.7%. Using multiple regression, it was determined that receiving praziquantel as treatment was associated with five factors: belonging to the Fulani or Moorish ethnic minority versus the Bambara/Soninke, use of the central versus the house-to-house drug distribution mode, the ratio of the population to the number of CDDs, the lack of supervision and belonging to the age group of 15 years or above (p<0.05). As well as that, it was found that the presence of parallel community-based programmes (HIV, tuberculosis) that provide financial incentives for community members discouraged many CDDs (who in most cases are volunteers) to participate in the CDI approach due to a lack of incentives.

**Conclusions:**

The findings indicate that the success of the CDI approach depends on, amongst other things, the personal characteristics of the respondents, as well as on community factors.

## Multilingual abstracts

Please see Additional file 1 for translations of the abstract into the six official working languages of the United Nations.

## Background

Schistosomiasis remains to be a major public health problem in many parts of Sub-Saharan Africa. It is estimated that, globally, there are 207 million people infected with one of the three major species of schistosomes [1], more than 90% of which occur in Sub-Saharan Africa [1,2]. A review of the relationship between infection and clinical morbidity in Sub-Saharan Africa estimates that deaths due to schistosomiasis may be as high as 200,000 per year [3]. In Mali, schistosomiasis is mainly associated with water resource development around dam construction. The prevalence rate of the disease varies from 40% in savannah villages to 80-90% in Office du Niger and Bandiagara [4-6].

For the past 20 years, strategies have been developed to control schistosomiasis in endemic areas. The key elements of these strategies were to control morbidity especially by selective treatment of heavy infections through regular treatment of high-risk groups (schoolchildren, fishermen and irrigation workers). It has been shown that mass treatment and health education of the population have significantly reduced transmission and morbidity in countries and regions such as Brazil, Venezuela, China, Indonesia, the Philippines, the Maghreb and the Middle East [7]. Despite this progress, schistosomiasis is far from being controlled in many parts of world. Because of the difficulties that exist in relation to access to treatment, as well as the non-involvement of communities in the process of the distribution and treatment of schistosomiasis, these strategies have failed to reach their objectives. Today, less than 10% of the treatment-eligible populations living in endemic regions of Africa, Asia and the Americas receive annual treatment for schistosomiasis, intestinal helminth infections and/or trachoma [2]. To improve access to treatment at affordable and sustainable costs and, where possible, to improve existing programmes, community-directed intervention (CDI) has been presented as an alternative strategy to control schistosomiasis and soil-transmitted helminths (STHs). This approach has been adopted in several settings [8-10] and has been successful in many countries as part of the African Programme for Onchocerciasis Control (APOC) [11,12], as well as for lymphatic filariasis control [13,14]. In Mali, despite the Schistosomiasis and Soil-transmitted Helminths National Control Programme (PNSHs) adopting the CDI approach, the programme has faced some difficulties which led to very low coverage (<60%) in some districts (oral communication of the PNLSH’s coordinator).

The overall objective of this study was to identify the prominent factors affecting the programme’s failure to improve access to treatment for all those at risk of both overt and subtle morbidity due to schistosomiasis in the health district of Diéma, Mali.

## Methods

### Experimental design and methods

#### Overall study design

The study focused on *i*) census and description of social and demographical data at baseline, *ii*) choice and selection of CDDs, *iii*) the social and cultural context of the CDI approach, and *iv*) evaluation of the study outcomes (treatment coverage, performance of CDDs, and involvement and perception of populations) one month after drug distribution started, and the issues related to side effects 24 hours after drug administration. The study was conducted between December 2007 and November 2008.

#### Sampling design and sample size

The study’s design was an exploratory and descriptive trial at the community level. The study unit, determined by the implementation level of the health services, was a village. The study population was comprised of an entire village population. To measure the success of this design, four villages were selected based upon the prevalence of schistosomiasis, the functionality of the health services and village accessibility. First, all functioning health areas in the 13 villages were listed. Ten villages were then selected from these health centres based on the accessibility and the acceptance of the population to participate in the study. The sample size of the eligible population (children five to 15 years of age who would provide urine and stool samples for analysis) was calculated according to the total population in each village, the precision (5%), the attended prevalence in the study site (60%) and the risk (alpha 5%) as described [15]. According to the statistical and demographical projection data, the population size in all ten villages was 9,806. However, to get more information on the population and the number of tablets required, the research team and the community drug distributors (CDDs) determined the study population after a new census was completed and the population of the villages was confirmed to be 8,022.

#### Study sites and health systems

In Mali, schistosomiasis is prevalent all over the country, but is endemic only in four (35 districts) of the eight regions. Following the Schistosomiasis Control Initiative (SCI), the CDI strategy was successfully implemented in the entire endemic district for schistosomiasis in Mali since 2005. However, the CDI strategy failed in the district of Diéma, in the north-west of Mali, where the coverage rate was lower than 60% [16]. Diéma was selected as a study site because of the CDI strategy’s failure to raise coverage, as expected, in this district compared to the other districts.

**Figure 1:**
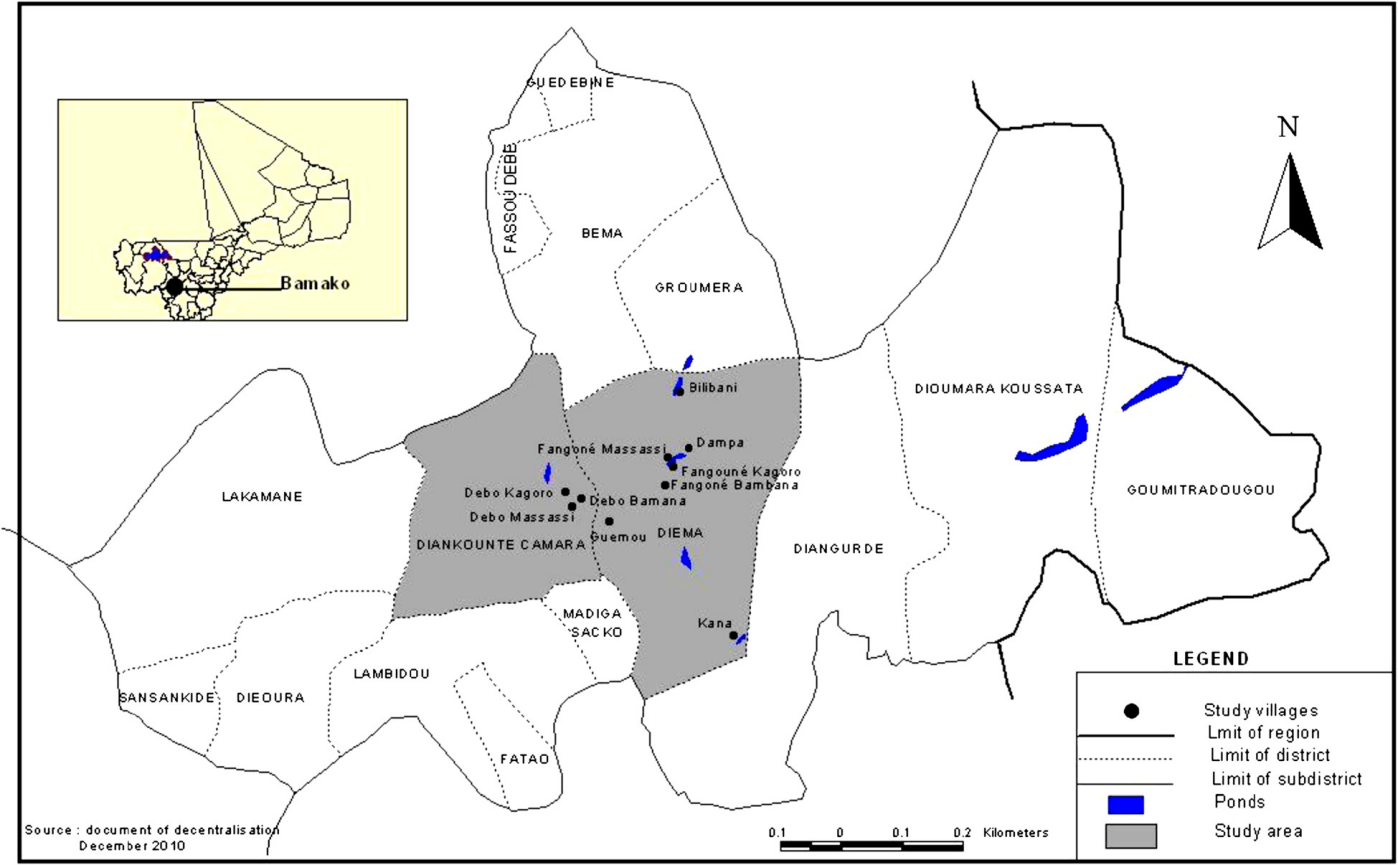
Localization of the ten study villages (*) in the district of Diéma, Mali.

The study took place in ten endemic villages in Diéma (Figure 1). All the villages are accessible by car and range in distance from seven to 20 kilometers from Diéma.

Health services in Diéma are organised in two levels: the community and the district level. Services at the community level are delivered by a primary health care centre (CHC). They are managed by selected community representatives and led by a health officer appointed by the government. The management of the district health services (DHS) is done by the district management team and headed by the District Medical Officer (DMO). The CHC management team defers to the DMO as the overall leader of the district health system. The access to health facilities varied from $0.4 to $1 per visit. In some villages, the health charges were sometimes evaluated into millet, rice, peanuts and/or poultry. Praziquantel (PZQ) is not always available at the health centre – it is only available during the mass drug distribution campaigns. Normally, patients suffering from schistosomiasis were examined (using questionnaires at the community level, or using a microscope for stool and urine examination at the district reference health centre). After routine examination, each patient would pay for the PZQ when he/she tested positive.

The social organisation of these communities was primarily hierarchical, based on lineage. The leadership includes a village head and the village council comprising of the heads of the village families. The village head and the council are responsible for the general administration and decision making of the village. At the community level, socio-economic activities are done according to sex and age groups. In some villages, because of the weight of tradition, women and men cannot mingle or attend meetings together. The illiteracy rate is generally high (70%) even if the policy of adult education in local languages is well developed. The main ethnic groups are Soninke and Bambara, and there are minority groups such as Fulani and Moorish. Islam is the major religion. The most common occupation is farming and cattle rearing, with close proximity to standing water sources. Young men in the region migrate extensively within, and outside, the country for economic purposes.

There are two major seasons: a rainy season from June/July to September/October, and a dry season which is divided into a cold season from November to January and a hot season from February to May. Schistosomiasis is seasonally transmitted from July to December.

### Techniques of the study

#### Choice and selection of community-directed health workers (CDHWs)

Community drug distributors (CDDs) were proposed for, and selected by, the population. One CDD per 125 inhabitants was selected and trained. Prior to the training of the CDDs, each team conducted health education using posters and/or verbal presentations in every CDI study village to address the following issues: knowledge of the disease, knowledge of treatment, attitude to treatment, attitude to disease, attitude to the distributor, attitude to good keeping and information/record sharing. Following the training, the CDDs were expected to be able to describe the major clinical manifestations of schistosomiasis, the different methods of treatment (house-to-house versus central point), and to have a working knowledge of the methods of carrying out the following PZQ distribution-related activities: community mobilisation, household enumeration, guidelines on the period and the duration of treatment, guidelines for the exclusion criteria, correct dosage using height, recognition of minor reactions and when to refer adverse reactions, basic record keeping, and to identify and report the issues encountered during the distribution. These guidelines could be modified when necessary. The dosage of PZQ for eligible members of the population was determined using a person’s height measurement [17]. An easy-to-transport stick was calibrated to serve as the measuring ruler. Tablets were swallowed in the presence of the distributor.

Attitude and perception of community-directed health workers (CDHWs) in the CDI strategy were scored on a scale ranging from strongly agree to uncertain to strongly disagree. Two major opinion items were considered to assess the health workers’ role in CDTI: *i*) the CDHWs would like to participate in the training of community members to take responsibility for PZQ and albendazole (ALB) distribution, and *ii*) the CDHWs would like to take part in all decision-making processes concerning the distribution of PZQ and ALB at the community level.

#### The social and cultural context of community-directed intervention (CDI)

The CDI strategy is an approach whereby community members collectively: (i) discuss a health or developmental challenge; (ii) design the approach to implement the interventions in the community; (iii) identify the resources to accomplish the task; and (iv) plan how, when, where and by whom it will be implemented. Like in the control of onchocerciasis [18], one aspect of the CDI strategy for schistosomiasis control through PZQ distribution is the appreciation and use of socio-cultural aspects of the communities, such as the social structures, legal system, resource mobilisation and sharing systems. Selection of many CDDs and CDHWs is vital for achieving high-drug coverage and integrating health programmes within the CDI strategy, respectively. Having, at least, both female and male CDHWs at the kinship level ensured prompt, equitable and quality healthcare delivery for all categories of community members. So, in some villages, females were not eligible to be CDDs due to the social and cultural context of that village.

In terms of why there was low morale amongst health workers and CDDs, most claimed to have been busy at the health units or at home, respectively, but many also had low morale because of their poor remuneration. However, it was not clear if CDHWs and CDDs, when involved in other health and development activities, could continue distributing PZQ effectively and efficiently, whether increased responsibilities would result in a higher drop-out rate and whether they would demand monetary incentives as a condition for services provided.

### Community mobilisation

During the first visit to a village, the research team met the village head or his representative to explain the purpose of the study. The research team met the entire community at a mutually convenient date. These meetings provided an opportunity to review the problem and transmission of schistosomiasis, and the benefits of controlling the morbidity. The community engaged in a discussion regarding their experience with the disease, snails, haematuria and systemic symptoms, such as abdominal pain. The conditions for receiving PZQ and ALB were outlined, procedures were reviewed for recognising exclusion criteria (less than four years of age and severely ill), and it was discussed that only eligible persons would be treated, that the individual doses would be determined by height and that possible side effects would be recognised.

### Demographical assessment

A census of the whole population in each village was performed at the onset of the study. The name of the village; the date of the census; the household identification number; the name of the head of the family; the name of the first wife followed by her children in order of decreasing age; and the sex, ethnicity, age and occupation of the other family members were recorded.

### Drug distribution

The CDDs identified those eligible for treatment at the regular scheduled time of PZQ distribution. Children who were less than four years of age or below 94 cm (observation or questioning) and seriously ill individuals were deemed ineligible. The CDDs selected by villagers went from house to house (house-to-house distribution mode) or chose a central place in the village (central distribution mode) for the drug distribution. People who could not be present were examined at the house of the distributor and administered the drug there. The two drugs, PZQ and ALB, are anthelminthic drugs used by the National Control Programme, and are safe and tolerable. The dose of PZQ was determined according to the height of the patient [16] and it was suggested that patients eat before taking it. The dose of ALB was one tablet for all those eligible whatever the age.

#### Treatment coverage determination

The target population was the eligible population of each village. All the treated and non-treated people were recorded by the CDDs in a register. The treatment coverage rate was calculated by dividing the number of people who received PZQ and ALB by the total population of the village. The geographical coverage was determined by ethnicity, geographical position of families in the village and the individual’s occupation.

#### Evaluation of the performance of the CDDs

The performance of the CDDs was assessed by randomly selecting interviewees from 10% of all households. All members of the sampled households were interviewed to determine if they received and were administered the drugs. In the event that no household members required therapy, explanations were recorded. The height of each individual was re-measured in order to crosscheck with the dose of the PZQ they received. People above 15 years of age were allowed to self-respond, however, for younger children, a guardian responded. The head of the household was also interviewed with respect to awareness of the drug delivery programme and its purpose.

### Evaluation of the community involvement and acceptability

A questionnaire was administrated to the heads, as well as to the councillors, leaders and healers, of each village. The questionnaire determined the success of each village in implementing the activities of the CDI: how they received the research team, how they mobilised their populations for the implementation of the CDI strategy, how they selected their CDDs (number and quality), and what resources they committed to support the activities of the CDI.

### Record keeping

Record keeping was standardised for all study villages. Data were collected in registry, except for interviews for which a guide was used. Each distributor had a list of all members of the villages. The following information was asked in the questionnaire: region, village, house number, name, sex, age, relationship to the head of the household, height, number of the eligible population, number of people treated, severe adverse reactions, number of tablets received, number of tablets distributed and remarks.

### The issues of adverse side effects

A side effect or incidence of a symptom is defined as a symptom absent before the treatment and experienced after the treatment. Amelioration of a symptom is defined as a symptom that was experienced before the treatment and was no longer present 24 hours after the treatment. To describe the side effects associated with PZQ administration, a questionnaire was administered immediately, and 24 hours, after treatment whereby children were asked whether they needed any medical assistance or felt any of the following symptoms: dizziness, headache, sleepiness, fatigue, vertigo, abdominal pain, cramps, nausea, vomiting, diarrhoea, bloody stools, lower back pain and urticaria/rash. Meanwhile, parents or teachers were also invited to respond on behalf of their children about whether they experienced any of the stated symptoms prior to the treatment and at 24 hours after the treatment. The CDDs were trained to recognise and manage any adverse events related to PZQ and ALB administration. In the case of serious adverse events, the CDDs were invited to refer the patients to the CHC.

#### Data processing and analysis

Each of the village CDDs entered all data on the population and the drugs administered. Quantitative data, including variables of acceptability, awareness of drug distribution and its purpose (by questionnaire administered to a sample of a household or their representative), the treatment coverage (characteristics of the respondents such as age, gender and ethnicity; design; number of distributors; method of CDDs’ selection; CDDs’ education and occupation; sex of the distributor; mode of distribution; and supervision) were entered into a computer and analysed using EPINFO 6.0 for trends and frequencies. A chi2 test was used for categorical variables, and logistic regression was used to test associations between factors and the variables they influence. P values less than 0.05 were considered to be significant.

Qualitative data from the process (sensitisation and planning of mass drug distribution, training and selection of CDDs, development of communication for social change (CFSC), drug procurement and distribution, and record keeping) were collected using observations notes, focus group discussions (FGDs) and in-depth interviews. They were transcribed and entered into a computer with TEXTBASE ALPHA. Segments were coded for thematic analysis of the process described above. The qualitative study in the form of descriptions, opinions or discussions was used to describe the beliefs and motivations that underline the access to treatment for all those at risk of schistosomiasis. The rationale of the qualitative study was to identify factors associated with the coverage rate (high and/or low rate) in order to improve the strategy. It also helped to explain or clarify findings from the analysis of the quantitative data. The qualitative study was carried out one month after the start of the drug distribution. It included a FGD and in-depth interviews.

A FGD lasting from one to two hours was led by a trained moderator. The FGD was held in five randomly-selected villages from the list of the study-site villages. In each village, ten households were randomly selected: one woman and one man (adults over 15 years) from each household (i.e., 20 respondents per village) were selected. Two groups for discussion (one for women and the other for men) were constituted. Each group was interviewed using a questionnaire aiming to capture respondents’ opinions, beliefs and motives.

The in-depth interviews were structured to establish attitudes, beliefs and knowledge, and were conducted in the five selected villages. Three series of interviews were held in each village: *i*) the village heads and leaders, *ii*) the distributor(s) or the person responsible for the PZQ distribution, and *iii*) the members (adults) of the ten households in the villages.

All data were presented in the form of narratives, frequency tables and histograms. Frequencies, means and standard deviations were calculated and included socio-demographic variables (gender, age, household size, etc.), socio-economic statuses, as well as others.

#### Ethical considerations

The proposal was reviewed and approved by the Institutional Review Board (IRB) of the Faculty of Medicine, Pharmacy and Dentistry at the University of Bamako. Community consent was obtained before starting the study. A consent form (or assent form for minors) was signed by each study participant.

## Results

### Treatment coverage of praziquantel and albendazole

A total of 8,022 people were studied in the survey, according to the community drug distributor (CDD) registry. At the end of treatment, the CDDs documented all eligible villagers who received medication. Based on the results recorded in Table 1, the mean coverage rate was 76.7% (6156/8,022) (range: 64.0% to 90.7%). Nearly 80% (78.2%) of school-age children were reached. Overall, 60% of the study villages had a coverage rate above 75%, with treatment coverage significantly differing between villages (p<0.01). The mean age of people who received praziquantel (PZQ) and albendazole (ALB) (24.5 years) did not significantly differ from those who did not require therapy (24.7 years). There were more females (52.2%) than males (47.8%), especially in the age group 15–34 years (p<0.01). However, the coverage did not vary between the sex (76.4% versus 76.3% for females and males, respectively). The pattern of treatment coverage did not vary significantly between sex among the different age groups (p>0.05) either, except for the 35–44 year old age group (p<0.01).

**Table 1:** **Treatment coverage per village** (% **in parenthesis**) **in the Diéma district**, **October 2008**

**Treatment villages**	**Total**	**No. treated**	**No. not treated**
Fangoune Kagoro	754	588 (78.0)	190 (24.3)
Fangoune Massassi	722	591 (81.9)	131 (18.1)
Fangoune Bambana	602	386 (64.1)	216 (35.9)
Dampa	810	665 (82.1)	145 (17.9)
Bilibani	487	351 (72.1)	136 (27.9)
Debo Massassi	884	714 (80.8)	170 (19.2)
Debo Bambana	1221	837 (68.6)	384 (31.4)
Debo Kagoro	478	388 (81.2)	90 (18.8)
Guemou	1005	675 (67.2)	330 (32.8)
Kana	1059	961 (90.7)	98 (9.3)
**Total**	8022	6156 (76.7)	1866 (23.3)

Analysis of the treatment coverage (those who took PZQ and ALB) by sex, age, ethnicity, mode of distribution and treatment ratio (population ration to CDDs) in eligible villagers (Table 2) showed that 78.1% of people under 15 years of age required medication compared with 75.3% of those above 15 years of age (p<0.01). There was a significant difference in compliance between ethnic groups: 77.3% of the 6,978 participants reported as belonging to the Bambara/Sarakolle group compared with 70.4% to the Fulani/Moorish group (p<0.01). In one village where the mode of distribution was centralised, 67.1% of the 1,005 eligible inhabitants received medication, compared to 78.3% of the 7,017 from villages with a house-to-house mode of distribution (p<0.01). The number of distributors per community ranged from one to seven. The ratio of the village population to CDDs was commonly associated with coverage (OR=3.40; IC= 2.73-4.24). In some villages, other available community members helped during the distribution stage. In nine villages, where 74.2% of 7,005 people received drugs, more than 250 people were treated per CDD. This is in contrast to two villages where only 150 people were treated per CDD; 90.7% of 1,059 received the drugs. It was also found that the presence of health workers for supervision at the time of distribution led to high coverage rates (p<0.01; OR=2.21 IC: 1.92-2.55).

**Table 2:** **Treatment coverage by sex**, **age**, **ethnicity**, **mode of distribution and treatment ratio** (**population to CDDs ratio**) **in eligible villages in the Diéma district**, **October 2008**

**Factor**	**No**	**Percent treated**	***p***	**OR**	**95% CI**
**Sex**					
Male	2984	76.4			
Female	4168	76.3	*0*,*97*	1.00	0.90-1.11
**Age group**					
< 15 years	3026	78.1			
15 and above	4996	75.4	<0.01	1.17	1.05-1.30
**Ethnicity**					
Bambara/Sarakolle	6978	77.3			
Fulani/Moorish	1044	70.4	<0.01	1.43	1.24-1.65
**Mode of distribution**					
Central	1005	67.2			
House-to-house	7017	77.7	<0.01	1.16	1.05-1.27
**Ratio of population to CDDs**					
150/CDD	1059	90.7			
>=150/CDD	6963	74.2	<0.01	3.40	2.73-4.24
**Presence of health workers**					
Health worker	1950	85.4			
No health worker	6072	73.4	<0.01	2.21	1.92-2.55

Multivariate analysis using logistic regression shows that a person’s ethnic group and age group, the mode of distribution, the population to CDD ratio and the presence of a health worker to supervise were all significantly associated with coverage rate increase (Table 3). Ethnic group and age group are personal characteristics of the respondents, while the mode of distribution, the population to CDD ratio and the presence of health workers at the time of distribution are community factors.

**Table 3:** **Factors associated with having received praziquantel and albendazole in the Diéma district**, **October 2008**

**Variables**	**OR**	***p***	**IC 95%**
**Sex**	0.97	0.67	0.87-1.08
**Ethnic group**	1.34	<0.01	1.16-1.55
**Age group**	1.13	0.03	1.01-1.26
**Mode of distribution**	1.45	<0.01	1.25-1.68
**Population to DCM ratio**	2.27	<0.01	1.74-2.97
**Presence of a health worker**	1.38	<0.01	1.16-1.66

**Figure 2:**
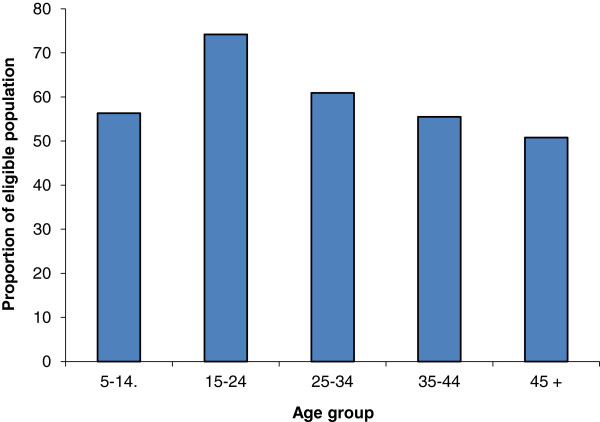
**Proportion of eligible people (by age group) who did not take praziquantel as they were absent, Diéma, October 2008.**

Overall, 23.3% (1,866/8,022) of the respondents did not receive medication at the time of treatment. From this group, 9% were excluded because they were underage. Another 11% were eligible, but were either sick, pregnant or unaware of the distribution (205/1,866). Only 1.7% refused to be treated. The pattern of absenteeism by age group showed that the problem was more common in those aged 15–24 years (Figure 2). Ethnic differences, however, did exist: only 2% of 297 Fulani and Moorish and 10% of 1,569 Bambara and Sarakolle complained that they had not been informed. Being absent at the time of distribution was more common among the Fulani and Moorish (70.3%) than the Bambara and Sarakolle (30.2%).

### Treatment and side effects

Side-effect data were available for 1,884 children and 1,640 mothers and teachers. During administration of the PZQ and ALB, side effects directly related to tablet administration (e.g., choking or coughing) were very rare (prevalence <0.1%) and not life threatening. In the ten villages, all side effects were investigated 24 hours after treatment. Most of the side effects were reported at varying prevalence levels, from 4.6% of children complaining about abdominal pain to 11.7% reporting fatigue. Headaches (5.1%), vomiting (9.7%) and diarrhoea (10.2%) were also found at significant levels. By contrast, mothers reported very few children (<1.0%) displaying any symptoms during the 24-hour follow-up.

## Discussion

In the Diéma health district, the influence of social behaviour and cultural beliefs on public health interventions was greater than expected, especially among women. This is evident in the majority of the CDDs being men because women and, in particular younger ones, were not accepted for this position. In some villages, the two sexes could not mingle at a single meeting place.

One of the main findings was that many CDDs dropped out early due to a lack of incentive that was being provided (it was felt that the work load was too heavy for no financial compensation). Regarding the community interaction with the health services, the majority of the health workers did not visit villages regularly. Only 40% of the population reported that the District Director of Services (DDS) visited their communities. For 60% of the population, health workers visited the villages only during a specific campaign, such as immunisation and/or a cholera outbreak. Of those interviewed, 70% reported that the services provided in the communities are relevant to their needs and that they were satisfied with them. In terms of the community having an understanding of schistosomiasis and intestinal helminths, and their experiences with the treatment, it was found that 98% knew about the disease and 46% felt that it is a problem mainly because children who have the disease are constantly ill. The disease aetiology was attributed partly to a child’s normal biological development (45%), food (30%) and contact with urine of those infected (20%). Over half (60%) of the population felt that schistosomiasis could be cured. Of those who believed it could be cured, ~50% thought that it could be treated with traditional medicine and the other half believed it could be done via modern medicine. With regards to prevention, 6% thought that the diseases caused by soil-transmitted helminths could be prevented, but no prevention was proposed for schistosomiasis.

Despite the health education about the transmission and prevention of the disease, standing water sources continued to be used by populations for fishing or for other domestic activities. After the rainy season, snails concentrate in small standing water sources with high levels of cercariae emerging. According to historical data, Diéma is known to be an endemic area for schistosomiasis haematobium [19] and our findings from the survey showed a high prevalence of *S*. *haematobium* (78.4%) (data not presented here). In Niger, re-infection begun five months after treatment and the initial prevalence of infection decreased from 74.5% to 47.1% [20].

The mean coverage rate, especially in school children, achieved under community-directed intervention (CDI) was higher than that recommended by the World Health Organization [5]. Since 2002, the World Health Assembly, WHA 54.19, requires that by 2010, any round of anthelminthic treatment must be offered to at least 75% of children aged six to 15 years. Experience in community-directed treatment (ComDT) of onchocerciasis [21,22] demonstrated that this treatment approach was not merely effective in the short term, but also proved to be sustainable in the long term. The success of the experience achieved in onchocerciasis led to extensions of ComDT to treatment of other disease control programmes, such as lymphatic filariasis [23] and schistosomiasis [8].

Though it is not yet known precisely what level of treatment coverage is required to achieve elimination of the disease, the coverage rate of 75%, especially in school-age children, may be sufficient to interrupt transmission. Experience of parasite control programmes has shown that 75% coverage is an attainable target, delivering significant reductions in morbidity and preventing irreversible sequelae. The results of the census showed that coverage was higher in females than in males. This may be a result of the intense migration among males between the ages of 15–34 years travelling within neighbouring countries (Senegal, Cote d’Ivoire) or Europe and America. The income from these migrant males contributed to more than 80% of family budgets. With respect to the treatment prevalence, males and females were equal (76%). Conversely, 15–29 year-old females in Cameroon were considered to be too busy with housekeeping activities and therefore less able to come to ivermectin distribution points [24]. However, in our study, the reasons for why eligible people did not receive PZQ were the same for males and females. The low coverage rate amongst 15-24-year olds (Figure 2) is often observed in this age group and tends to be related to economical concerns of this mobile population.

Absenteeism at the time of distribution was more common among Fulani and Moorish populations (70.3%) who were primarily migrant cattle breeders compared to young Bambara/Sarakolle (30%). The fact that some people (Fulani, Moorish and some resident Bambara/Sarakolle) were absent from villages at the time of distribution may be of epidemiological importance. These people, as well as migrants, remain to be important parasite reservoirs and may re-infect snails by shedding schistosome eggs upon return to the village. The Fulani and Moorish present a very different case. They live in separate settlements near the villages and they do not integrate within the Bambara and Sarakolle farm hamlets. They herd cattle in order to find pasture land, which explains why absenteeism at the time of distribution was a common reason for not receiving PZQ and ALB.

To meet CDI objectives, CDDs and health workers may need to pay more attention to ethnic diversity in order to build specific treatment schedules according to ethnic presence in the village. Even if the house-to-house mode distribution appeared to be efficient in this study, the success of this approach to increase coverage depends on the number of CDDs and their involvement in all aspects of distribution. Qualitative data helped to explain some findings. The following FGD comments reveal how villagers perceived drug distribution. Some said ‘that the drug would affect them’. ‘It makes [me] giddy. I didn’t use [it] because of side effects’. Some individuals who were unable to be present for the drug distribution reported that they refused to take the drugs after hearing about their side effects. ‘It causes stomach aches, vomiting and dizziness for those who use it. Some of them cannot work.’ Overall, side effects occurred in 12.3% of the eligible participants (dizziness – 5.9%, nausea, vomiting – 5.5% and others – 0.9%). Some individuals initially rejected therapy but later took it ‘because they got information from others that the drug was good’. ‘It works.’

In-depth interviews with the Fulani or Moorish identified some of the reasons why their coverage rate was lower. ‘We are migrants and most of us are following flocks and herds outside the village.’ The native villagers, the Bambara and Sarakolle, confirmed the problem during the FDGs. ‘At the time of the distribution, even if the Fulani and Moorish were present at hamlets (two to four kilometres from village), they did not move to the village for treatment but preferred to be treated in their own gomes’.

The role of community participation in improving coverage was identified by the CDDs during the FGDs. The comments by the CDDs distinctly differed to the present coverage status. The comments from those villages with low coverage were more negative: the community members were not involved in any other form of treatment besides the drug they took, they did nothing to assist during the distribution and the community did not cooperate as expected. ‘I did not get any kind of assistance from anybody in carrying out the distribution.’

In high coverage villages, the following positive comments from the CDDs were common: ‘the community members assisted in the area of health education, mobilisation and procurement of census materials; the community showed an interest in the programme as they helped me on the farm; community members brought a notebook for the census and a drug box; they fetched water when we were using the drug; they helped me to hold stick when I was distributing the drug’.

Besides these comments by the CDDs, village leaders, during the FGDs, typically gave the following comments, especially in low-coverage villages: ‘In our village, most of the CDDs refused drug distribution because they prefer to be compensated for their participation, such as being noted in other programmes (i.e., HIV). Out of every five to six CDDs at baseline, only one or two of them finally accepted to do the job’. Sometimes, various programmatic communication problems were identified by the leaders and community members: ‘On the distribution day, many residents were not around. Some men, and especially eligible children, took out their cattle for grazing’.

In high coverage villages, the role of the CDDs was judged to be positive: the CDDs showed an interest in the programme because their goal was to distribute drugs to all the villagers. When some community members were absent on the date of distribution, the CDDs returned twice or three times to the same family to meet them or waited for a period of time before returning to administer the drug. Even if people showed side effects, the CDDs encouraged them to use the drug, reassuring the villagers that the problems will eventually disappear. In fact, going from house to house was the best thing because they were able to reach even those who cannot walk. This was effective as people felt this was not interfering with their daily duties and because the CCDs were people they knew, they thus had faith in them.

## Conclusion

The aim of the CDI approach was to provide better coverage rates, or at least to get them to be as good as the traditional programme-designed strategies (and also to ensure sustainability of long-term programmes). The findings of this study demonstrated that participatory methods could be incorporated in an effective control strategy for distribution of PZQ and ALB for treatment of schistosomiasis and intestinal helminthiasis. The most important factor for successful implementation of the CDI approach was primarily community mobilisation in order to get a high number of distributors devoted to the process. In all villages, community distributors would appreciate some form of incentive or compensation. More attention to the personal characteristics of the respondents and to community factors may also help raise the coverage rates.

## Abbreviations

ALB: Albendazole; APOC: African Programme for Onchocerciasis Control; CHC: Community health centre; CDD: Community drug distributors; CDHWs: Community-directed health workers; CDI: Community-directed intervention; CFSC: Communication for social change; CI: Confident interval; ComDT: Community-directed treatment; DDS: District director of services; DHS: District health services; DMO: District medical officer; FGD: Focus group discussion; HIV: Human immunodeficiency virus; IRB: Institutional review board; OR: Odds ratio; PNLSHs: National Control Programme for Schistosomiasis and Soil-transmitted Helminths; PZQ: Praziquantel; WHA: World health assembly.

## Competing interests

The authors declare that they have no competing interests.

## Authors’ contributions

DA participated in the conception and design of the study, data analysis and interpretation. He also contributed to the writing of the manuscript and assured the coordination of the trial. He has also reviewed the final version. BB participated in the design of the study and in onsite execution by collecting and analysing data. He also had all the clinical responsibility, contributed to the drug distribution and coordinated the field activities. KB participated in the conception and in onsite execution, assessment of side effects, data analysis and interpretation. He also supervised drug distribution, and contributed to the writing of the manuscript and in data analysing. SO participated in data collection, helped draft the manuscript, and analysed and reviewed the final version. DO participated in the conception and design of this paper. He contributed to the writing of the manuscript and the data analysis, participated in drafting of the paper and reviewed the final version. All authors read and approved the final manuscript.

## Supplementary Material

Additional file 1 Translation of the abstract into the six official working languages of the United Nations. Click here for file
