# Supplementary material for: Factors associated with coverage of praziquantel for schistosomiasis control in the community-direct intervention (CDI) approach in Mali (West Africa)
Source: Infect Dis Poverty. 2013 Jun 10;2:11. doi: 10.1186/2049-9957-2-11 (PMC3710142; doi:10.1186/2049-9957-2-11)
Supplement: Additional file 1 — Translation of the abstract into the six official working languages of the United Nations. [file 2049-9957-2-11-S1.pdf]

Translation of the abstract into the six official working languages of the United Nations

العوامل المصاحبة لاستعمال برازيكوانتل لعلاج المنشقات في إطار التدخل الموجّه للمجتمع في مالي (غرب إفريقيا)

دابو أ، باري ب، كوريبا ب، سانكاريي أو، دوومبو أو

الملخص

الخلفية: بالرغم من التقدم الذي تحقق في السيطرة على الأمراض المدارية المهملة (**NTD**)، إلا إن بعض الحالات كالبلهارسيا (المنشقات) والعدوى بالديدان التي تنتقل عن طريق التراب ما تزال بعيدة عن المعالجة الفعالة في عديد من أنحاء العالم. العلاج الكيميائي، وهو العنصر الأساسي في كافة خطط السيطرة على هذه الأمراض، يواجه بعض الصعوبات المتعلقة بالحصول على العلاج. تهدف الدراسة التي نجريها إلى وصف العوامل التي تلعب دورا في نجاح أو فشل أسلوب التدخل الموجّه للمجتمع عن طريق برامج التحكم بالأمراض، والتي تهدف إلى تحقيق تغطية عالية متسقة مستدامة وبكلفة ميّسرة في المناطق التي تتوطن فيها هذه الأمراض.

الطرق: عند إدراج المرضى، أجريت مقابلة لكل طفل وأخضع لفحص طبي. تم البدء بتطبيق برنامج التدخل الموجّه للمجتمع منذ ديسمبر 2007 وحتى أكتوبر 2008 في عشر قرى في مقاطعة دييما، مالي. وقد ركزت الدراسة على:جمع البيانات، علاج الأفراد المؤهلين لتلقي العلاج، تقييم التغطية العلاجية، أداء موزعي الدواء من المجتمع المحلي (**CDDs**)، ومشاركة السكان وتفهّمهم.

النتائج: أجريت الدراسة على 8022 فردا من المؤهلين لهذه الدراسة بمعدّل تغطية وصل إلى 76.4%. وباستخدام الانحدار المتعدد تم التوصل إلى القرار بأن تلقي برازيكوانتل كعلاج ترافق بخمسة عوامل: الانتماء إلى أقليات الفولاني أو المغاربية (موريش) مقابل أقليات بامبارا/سونيكي، واعتماد نظام التوزيع المركزي للدواء مقابل التوزيع من بيت إلى بيت، نسبة السكان إلى موزعي الدواء المحليين، ونقص الإشراف والانتماء إلى المجموعة العمرية 15 سنة وما فوق (عامل الاحتمالية □0.05). وبالإضافة إلى ذلك، وجد أن الوجود الموازي لبرامج مجتمعية أخرى (نقص المناعي البشري المكتسب، والسل) تقدم حوافز مالية لأفراد المجتمع قد ثبطت مشاركة العديد من موزعي الدواء المحليين، الذين كانوا في معظم الحالات يعملون كمتطوعين في البرامج الموجهة للمجتمع، بسبب انعدام الحوافز.

الاستنتاج: تشير الموجودات إلى أن نجاح أسلوب التدخل الموجه للمجتمع يعتمد، من بين عوامل أخرى، على الصفات الشخصية للمتلقين، إضافة إلى عوامل مجتمعية.

Translated from English version into Arabic by Lina SM, through

TRANSLATORS WITHOUT BORDERS

西非马里使用吡喹酮防治血吸虫病的社区干预措施覆盖率的相关因素

Dabo A, Bary B, Kouriba B, Sankaré O, Doumbo O

摘要

引言：尽管在防治被忽视的热带病方面已取得了一定的进展，但是在世界上很多地方血吸虫和土源性线虫感染远未得到有效控制。化疗，这个所有防治策略的关键要素，在治疗的可及性上面临着一些困难。本研究的目的是描述防治项目中社区干预（CDI）措施的成功或失败的相关因素，进而在流行区实现负担得起和可持续的高覆盖率。

方法：所有孩子均接受采访，并进行体检。2007年12月至2008年10月，在马里Diéma区的10个村庄采用CDI方法进行防治。研究主要集中在：数据收集、符合条件人群的治疗、治疗覆盖率的评估、社区药品分发员（CDD）的表现，以及人群的参与和认知。

结果：共有8022人符合参与研究的条件，平均覆盖率为76.4％。利用多元回归分析，确定了与接受吡喹酮治疗相关的5个因素：归属Fulani或Moorish族群对Bambara/Soninke族群，中心分发药品模式对逐户分发药品模式，人群与CDDs的比例，缺乏监督和15岁或以上年龄组（$P<0.05$）。同时，发现同时开展的以社区为基础的项目（艾滋病，结核病）为社区成员提供财政奖励，这使许多CDDs因缺乏奖励而感到沮丧，因为在大多数情况下，这些CDDs是志愿者。

结论：研究结果表明，CDI的成功更多地取决于受访者的个人特点和社区因素。

Translated from English version into Chinese by Yang Pin, through

CHINESE CENTER FOR DISEASE CONTROL AND PREVENTION
NATIONAL INSTITUTE FOR PARASITIC DISEASES

Les facteurs associés à la couverture en intervention sous directives communautaires (CDI) avec le praziquantel comme stratégie de lutte contre la schistosomiase au Mali (Afrique de l'Ouest)

Dabo A, B Bary, Kouriba B, Sankaré O, Doumbo O

Résumé

Contexte: Malgré les progrès réalisés dans la lutte contre les maladies tropicales négligées (MTN), les schistosomes et les infections dues au helminthes transmis par le sol sont loin d'être gérés de façon efficace dans de nombreuses régions du monde. La chimiothérapie, l'élément clé de toute stratégie de contrôle, est confrontée à des difficultés en termes d'accès au traitement. Notre étude vise à décrire les facteurs impliqués dans la réussite ou l'échec d’une stratégie d’intervention sous directives communautaires (CDI) à travers des programmes de contrôle, qui visent à atteindre une couverture élevée, constante, à des coûts abordables et durables dans les zones endémiques.

Méthodes: A l'inclusion, chaque enfant a été interrogé et soumis à un examen physique. La stratégie CDI a été réalisée à partir de Décembre 2007 jusqu’à Octobre 2008, dans une dizaine de villages du district de Diéma, au Mali. L'étude a porté sur: la collecte des données, le traitement de la population éligible, l'évaluation de la couverture du traitement, la performance des distributeurs communautaires de médicaments (CDD), l'implication et la perception des populations.

Résultats: Un total de 8.022 personnes admissibles ont été étudiées avec un taux de couverture moyen de 76,4%. En utilisant la régression multiple, il a été déterminé que l’administration du traitement praziquantel a été associée à cinq facteurs: l'appartenance à des minorités ethniques Peuls ou Maures versus Bambaras / Soninkés, l’utilisation de la centrale par rapport au mode de distribution des médicaments de maison en maison, le ratio de la population par rapport au nombre de CDD, le manque de supervision et appartenance au groupe d'âge des 15 ans ou plus ($p < 0{,}05$). En plus de cela, il a été constaté que la présence de programmes communautaires parallèles (VIH, tuberculose) qui fournissent des incitations financières aux membres de la communauté ont découragé de nombreux CDD, (qui dans la plupart des cas sont des bénévoles), à participer à la stratégie CDI en raison d'un manque de incitation.

Conclusion: Les résultats indiquent que le succès de la stratégie CDI dépend, entre autres des caractéristiques personnelles des répondants, ainsi que des facteurs communautaires.

Translated from English version into French by Nadinetrans, through

TRANSLATORS
WITHOUT BORDERS

Факторы, связанные с охватом населения при лечении празиквантелом для контроля шистосомоза методом направленного общиной вмешательства в Мали (Западная Африка)

Дабо А, Бари Б, Куриба Б, Санкарэ О, Думбо О

Аннотация

Краткое описание: Несмотря на прогресс в борьбе за контроль забытых тропических болезней, во многих частях мира далеко не всегда эффективно справляются с заболеваниями шистосомозом и геогельминтом. Контроль с помощью ключевого элемента всех стратегий химиотерапии сталкивается с определенными трудностями из-за доступа к лечению. Задачей нашего исследования является описание факторов, связанных с успехом или неудачей применения программ направляемого общиной вмешательства (CDI), нацеленных на достижение стабильно высокого охвата при доступных и устойчивых ценам в эндемических районах.

Методы: При включении проводился опрос и медицинский осмотр всех детей. Метод CDI применялся с декабря 2007 года по октябрь 2008 года в десяти деревнях округа Дьема, Мали. Исследование было направлено на сбор данных, лечение соответствующего критериям населения, оценку охвата медицинским обслуживанием, работы общественных распространителей лекарств, а также вовлечения и восприятия людей.

Результаты: Всего было обследовано 8022 человека, соответствущих требованиям включения, со средним показателем охвата 76,4%. При помощи множественной регрессии было установлено, что получение лечения празиквантелом связано с пятью факторами: принадлежность к этническим меньшинствам фулани или мавров в сравнении с бамбара/сонинке, централизованное распространение лекарств по сравнению со способом от одного дома к другому, соотношение численности населения и количества распространителей лекарств, недостаточный надзор и принадлежность к возрастной группе от 15 лет и старше ($p<0.05$). Кроме того, было выявлено, что наличие параллельных программ для сообществ с тем или иным заболеванием (ВИЧ, туберкулёз), которые материально поощряют членов этих сообществ, из-за недостатка стимулов не способствует участию в методе CDI многих общественных распространителей лекарств, большинство которых является добровольцами.

Заключение: Результаты показывают, что успех метода CDI зависит, помимо прочего, от индивидуальных особенностей респондентов и от общинных факторов.

Translated from English version into Russian by Natalia Potashnik, through

TRANSLATORS
WITHOUT BORDERS

Factores asociados con la cobertura en la intervención dirigida por la comunidad (IDC) con praziquantel para el abordaje del control de la esquistosomiasis en Mali (África occidental)

Dabo A., Bary B., Kouriba B., Sankaré O., Doumbo O.

Resumen

Antecedentes: A pesar del avance logrado en el control de las enfermedades tropicales desatendidas (ETD), en muchos lugares del mundo queda mucho por hacer para controlar eficazmente las infecciones producidas por helmintos esquistosomas y transmitidos por el suelo. La quimioterapia, elemento clave de todas las estrategias de control, se enfrente a diversas dificultades relacionadas con el acceso al tratamiento. El objetivo de nuestro estudio es describir los factores que intervienen en el éxito o el fracaso del abordaje la intervención dirigida por la comunidad (IDC) a través de programas de control, cuyo objetivo es lograr una alta cobertura consistente a un coste asequible y sostenible en áreas endémicas.

Métodos: En el momento de la inclusión, se entrevistó a cada niño y luego se le realizó el examen físico. El abordaje de IDC se realizó desde diciembre de 2007 hasta octubre de 2008 en diez pueblos del distrito de Diéma, en Mali. El estudio se centró en: recolección de datos, tratamiento de la población elegible, evaluación de la cobertura del tratamiento, desempeño de los distribuidores de fármacos de la comunidad (DFC) y la participación y percepción de las poblaciones.

Resultados: Se estudió a un total de 8 022 personas elegibles con un índice de cobertura promedio del 76,4 %. Utilizando la regresión múltiple, se determinó que recibir praziquantel como tratamiento se asoció con cinco factores: pertenecer a la minoría étnica Fulani o Moorish frente a la Bambara/Soninke, usar el modo de distribución central de fármacos en lugar del modo casa en casa, la proporción entre la población y la cantidad de DFC, la falta de supervisión y pertenencia al grupo etario de 15 años o mayor ($p<0,05$). También se halló que la presencia de programas paralelos basados en la comunidad (VIH, tuberculosis), que proporcionan incentivos financieros a los miembros de la comunidad, desalentaban a muchos DFC, que en la mayoría de los casos son voluntarios, a participar del abordaje de IDC por la falta de incentivos.

Conclusión: Los hallazgos indican que el éxito del abordaje de IDC depende, entre otras cosas, de las características personales de los entrevistados y de los factores de la comunidad.

Translated from English version into Spanish by Andrea Ali, through

TRANSLATORS
WITHOUT BORDERS
